# Development of a pan-genotypic monoclonal antibody-based competitive ELISA for the detection of antibodies against Bovine viral diarrhea virus

**DOI:** 10.3389/fimmu.2024.1504115

**Published:** 2024-11-25

**Authors:** Shuhui Qi, Jing Wang, Ting Le, Chao Sun, Jitao Chang, Zhigang Jiang, Xin Yin, Quanhai Pang

**Affiliations:** ^1^ College of Veterinary Medicine, Shanxi Agricultural University, Taigu, China; ^2^ State Key Laboratory for Animal Disease Control and Prevention, Harbin Veterinary Research Institute, Chinese Academy of Agricultural Sciences, Harbin, China; ^3^ Molecular Biology, Teaching and Research Center, University of Liège, Gembloux, Belgium; ^4^ Institute of Western Agriculture, Chinese Academy of Agricultural Sciences, Changji, China

**Keywords:** bovine viral diarrhea virus (BVDV), competitive ELISA (cELISA), BVDV E2, BVDV-1, BVDV-2

## Abstract

**Introduction:**

Bovine viral diarrhea virus (BVDV), a positive-sense single-stranded RNA virus, causes significant economic losses in the cattle industry. Current diagnostic methods for BVDV exhibit variable sensitivity and specificity, underscoring the need for more rapid and accurate detection approaches. Here, we developed a novel competitive ELISA (cELISA) to detect antibodies against the BVDV E2 protein.

**Methods and results:**

We generated three monoclonal antibodies (mAbs)—3E6, 2D5, and 5B9—by immunizing mice with purified BVDV E2 protein expressed in Expi293F cells. Among these, mAb 3E6 displayed superior competitive binding abilities to the E2 protein, enabling effective differentiation between BVDV positive and negative sera. Remarkably, mAb 3E6 exhibited pan-genotypic recognition of various BVDV strains, including BVDV-1a, -1b, -1c, -1m, -1p, -1v, and -2a, while showing no cross-reactivity with the classical swine fever virus (CSFV). Computational modeling using AlphaFold 3 identified domain B of the E2 protein as the primary binding site for mAb 3E6. Building upon these findings, we established a cELISA employing mAb 3E6 and recombinant E2 protein. Receiver-operating characteristic (ROC) analysis revealed outstanding diagnostic performance, achieving a sensitivity of 99.26% and specificity of 98.99%. Further tests confirmed the cELISA's specificity for detecting BVDV-specific antibodies, with no cross-reactivity with antisera from animals infected or immunized against BCoV, BHV-1, BRV, AKAV, LSDV, BLV, and CSFV. Consistency was observed between results from the BVDV E2 cELISA and traditional virus neutralization test (VNT), demonstrating high sensitivity for monitoring antibody dynamics. In performance evaluations, the established cELISA exhibited high concordance with VNT in assessing 160 vaccinated sera and 190 clinical samples.

**Discussion:**

The BVDV E2 cELISA, utilizing mAb 3E6 to target domain B of the BVDV E2 protein, represents a reliable and effective serological diagnostic tool for the detection of antibodies against both BVDV-1 and BVDV-2. This methodology holds significant promise for applications in clinical diagnosis and the evaluation of vaccine efficacy.

## 1 Introduction

Bovine viral diarrhea virus (BVDV), a positive-sense single-stranded RNA virus that belongs to the *Pestivirus* genus of the *Flaviviridae* family, is an important pathogen affecting cattle worldwide, leading to substantial economic losses (1, 2). Studies indicated that the seropositivity rate exceeds 80% in regions such as Australia, New Zealand, and Brazil, with production losses estimated up to $687.8 per affected animal. This scenario poses a serious threat to the sustainable development of the global ruminant breeding industry (3, 4). BVDV not only affects cattle of all ages but also poses a significant threat to other *Artiodactyla* species, including sheep, pigs, camels, and goats (5–7). Additionally, BVDV exhibits multi-tissue tropism in infected animals, causing persistent diarrhea, mucosal ulcers, reproductive disorders, and other clinical symptoms (8, 9). This virus consists of two biotypes: cytopathic (CP) and non-cytopathic (NCP) (10). While CP strains can lyse cells *in vitro* and lead to severe disease such as mucosal disease (3), NCP strains, which are more prevalent, may not present overt clinical symptoms (11). BVDV infections during the first trimester may result in fetal death, or the birth of persistently infected (PI) calves, which serve as major reservoirs for viral transmission and contribute to ongoing epidemic challenges (12).

The BVDV genome consists of a single open reading frame (ORF) flanked by two untranslated regions (UTRs). The ORF encodes a precursor polyprotein of approximately 4,000 amino acids. The polyprotein is further processed both co-translationally and post-translationally by viral and cell-encoded proteases to yield 11 mature viral proteins: N^pro^, C, E^rns^, E1, E2, P7, NS2-3, NS4A, NS4B, NS5A, and NS5B (13). Of these, the capsid protein (C) and three envelope glycoproteins-E^rns^, E1, and E2-are structural proteins involved mainly in the assembly of viral particles (14). The 5’UTR is the most used genomic sequence for phylogenetic analysis and BVDV genotyping, followed by sequences from N^pro^ and E2 (15). According to the Flaviviridae Study Group of the International Committee on Taxonomy of Viruses (ICTV), BVDV is categorized into three main genotypes: *Pestivirus* A (BVDV-1), *Pestivirus* B (BVDV-2), and *Pestivirus* H (BVDV-3) (16). BVDV-1 is further divided into 23 subtypes (1a-1w), BVDV-2 is divided into 4 subtypes (2a-2d), and BVDV-3 is divided into three sources: Brazilian, Thai, and Italian (17–19). The genetic diversity of BVDV, particularly in the E2 glycoprotein, presents challenges for immune response and diagnostic accuracy (10).

Rapid and accurate diagnosis of BVDV infection is critical for effective management and control of the disease within the cattle industry. Several diagnostic methods, including etiological, serological, and molecular approaches, are employed to detect BVDV, each offering specific advantages and disadvantages (20). Virus neutralization test is currently considered as the “gold standard” for diagnosing BVDV due to its high accuracy and straightforward methodology (21). However, it is time-consuming, and labor-intensive, with results often delayed (20). Reverse transcription polymerase chain reaction (RT-PCR) detection method is sensitive and rapid, but the PCR cannot accurately grasp the sampling time of acute BVDV infection and detect past infections, so it is difficult to evaluate the infection history of the group, and it requires complex equipment (22). Enzyme-linked immunosorbent assay (ELISA) serves as an essential tool in serological testing, differentiating between various antigen and antibody targets (23). Currently, the ELISA methods for detecting BVDV include the blocking ELISA (bELISA) or competitive ELISA (cELISA), which uses the NS3 protein as the recognition antigen. However, these methods are not suitable for detecting antibodies following vaccination (24, 25), especially for evaluation of the BVDV E2 subunit vaccines (26). Therefore, for herds vaccinated with inactivated BVDV vaccines, E2 subunit vaccines, or mRNA vaccines, it is recommended to utilize neutralization assays that primarily detect antibodies against the E2 protein to evaluate vaccine efficacy, as the E2 protein is a primary target for neutralizing antibodies that provide protection against BVDV infection. An alternative bELISA that uses the BVDV E2 protein as the antigen to measure antibody titer demonstrates strong specificity, with a diagnostic specificity of 96.43% and high sensitivity of 95.6%. Nonetheless, this method is effective only for diagnosing BVDV-1 and does not have a clear effect on other BVDV subtypes (27).

In this study, we generated a pan-genotypic monoclonal antibody (mAb) 3E6 that specifically recognizes the BVDV E2 protein and established a robust cELISA for the detection of antibodies against both BVDV-1 and BVDV-2. We then assessed the sensitivity and specificity of the developed cELISA, and compared its diagnostic performance with that of the traditional VNT. Our results demonstrated that the cELISA based on mAb 3E6 effectively detected antibodies against various genotypes of BVDV-1 and BVDV-2, showing its potential as a reliable tool for clinical diagnosis and antibody evaluation after vaccination.

## 2 Materials and methods

### 2.1 Cells and cell culture

Expi293F cells were maintained in the laboratory and cultured in 293Pro^®^ CD 293 M Serum-Free Medium (H731KJ, Basal Media). Madin-Darby Bovine Kidney (MDBK) cells and Porcine Kidney (PK-15) cells were also preserved in the laboratory and cultured in a custom medium consisting of Dulbecco’s Modified Eagle’s Medium (DMEM) supplemented with 10% heat-inactivated fetal bovine serum (FBS), 6 mM L-glutamine, penicillin (100 U/mL), and streptomycin (100 μg/mL).

### 2.2 Plasmids, virus and serum samples

pCAGGS-IL10(SP)-6 × His vector, BVDV-1b, BVDV-1c, BVDV-1v, BVDV-1m, BVDV-1p strains, and the CSFV clinical isolate were stored in our laboratory. The infectious clone BVDV-1a NADL-mCherry was provided by Dr. Diego E. Alvarez. BVDV-2a was a gift from Dr. Mingchun Gao at Northeast Agriculture University. The sera from pestivirus CSFV was provided from Dr. Huaji Qiu at Harbin Veterinary Research Institute. Positive sera for BCoV, BHV-1, BRV, AKAV, LSDV, and BLV were obtained from infected or immunized cattle.

### 2.3 Reagents and antibodies

The following reagents and antibodies were used in this study: ExpiFectamine^™^ 293 Transfection Kit (A14524, ThermoFisher Scientific, USA), High Affinity Ni-NTA Resin (L00250, GenScript, China), LE buffer (50 mM NaH_2_PO_4_, 300 mM NaCl), QuickBlue Rapid Glue Dye (BF06152, Biodragon), Polyethylene glycol/dimethyl sulfoxide solution (P7306, Sigma, USA), Hypoxanthine aminopterin thymidine (HAT) Media Supplement (50×) Hybri-Max^™^ (H0262, Sigma, USA), Protein G Resin (L00209, GenScript, China), Monoclonal Antibody Isotype Identification Enzyme Ready-to-Use Kit (BF16002X, Biodragon, China). The primary antibodies used in this study included 6 × His, His-Tag mAb (1:25000, 66005-1-Ig, Proteintech, China), bovine viral diarrhea virus type 1&2 (BVDV-1&2) MAb E2 gp53 IgG2b isotype (1:500 for WB, 1:1000 for IFA, 348, VMRD, USA), and bovine viral diarrhea virus (BVDV) antiserum (1:2000 for WB, 1:1600 for IFA, PAB-BVD, VMRD, USA). Secondary antibodies included Horseradish peroxidase (HRP)-labeled goat anti-mouse IgG (Fc specific) (1:5000, A2554, Sigma, USA), goat anti-mouse IgG (H+L) highly cross-adsorbed secondary antibody, Alexa Fluor^™^ 488 (1:1000, A-11029, Invitrogen, USA), and goat anti-pig FITC labelled antibodies (1:1000).

### 2.4 Recombinant BVDV E2 expression and purification

The BVDV E2 gene (with a deletion of 30 amino acids at the C-terminus) was amplified from the BVDV-1a NADL-mCherry strain and cloned into pCAGGS-IL10(SP)-6 × His vector at the *Nhe* I and *Kpn* I restriction sites. The recombinant plasmid was transfected into Expi293F cells, and after 5 days, the supernatant was collected. This supernatant, containing the BVDV E2 protein, was loaded onto Ni^2+^-nitrilotriacetate affinity (NTA) resin and washed with LE buffer containing 40 mM imidazole. The BVDV E2 protein was then eluted using LE buffer with 250 mM imidazole and dialyzed overnight at 4°C in 0.02 M PBS. The expression and purification of the BVDV E2 protein was confirmed by sodium dodecyl sulfate-polyacrylamide gel electrophoresis (SDS-PAGE) and by western blot using a commercial 6 × His, His-Tag mAb, a bovine viral diarrhea virus type 1&2 (BVDV-1&2) MAb E2 gp53 IgG2b isotype, and bovine viral diarrhea virus (BVDV) antiserum.

### 2.5 Production of BVDV E2 mAbs

Mice were immunized with the BVDV E2 protein on days 0, 21, and 42, for a total of three immunizations. One week after the final boost, the mice were euthanized to collect splenocytes. SP2/0 myeloma cells were fused with the splenocytes using polyethylene glycol and cultured in RPMI-1640 medium supplemented with 20% FBS and 1 × Hypoxanthine aminopterin thymidine (HAT). The cell suspension was dispensed into 96-well plates and incubated at 37°C with 5% CO_2_. Supernatants from the fused cells were screened using indirect ELISA (iELISA) with BVDV E2 as the coated antigen. Positive hybridomas from confluent wells were subcloned three times by limiting dilution to obtain single hybrid cell lines. Subsequently, the amplified hybridomas (2 × 10^6^ cells) were injected intraperitoneally into BALB/c mice pre-treated with liquid paraffin one week earlier. After seven days, mouse ascites was collected, and the anti-BVDV E2 mAbs were purified using Protein G Resin. The purified mAbs were analyzed by SDS-PAGE, and their subclass and light chain type were determined using a commercial Monoclonal Antibody Isotype Identification Enzyme Ready-to-Use Kit (Biodragon).

### 2.6 Indirect immunofluorescence assay

MDBK and PK-15 cells were seeded in 96-well plates at a density of 1×10^4^ cells per well. MDBK cells were infected with different BVDV strains, including BVDV-1a, BVDV-1b, BVDV-1c, BVDV-1m, BVDV-1p, BVDV-1v, and BVDV-2a, while PK-15 cells were infected with the CSFV isolate. After infection, the cells were fixed with 4% paraformaldehyde for 30 min at 4°C and permeabilized using 0.2% Triton ×100. Following a 1% BSA at 4°C overnight, MDBK cells were incubated with either a bovine viral diarrhea virus type 1&2 (BVDV-1&2) MAb E2 gp53 IgG2b isotype or mAb 3E6. PK-15 cells were incubated with either anti-CSFV serum or mAb 3E6. The next day, cells were washed with PBS and incubated with secondary antibody-goat anti-mouse IgG (H+L) highly cross-adsorbed secondary antibody, Alexa Fluor^™^ 488 or goat anti-pig FITC-for 45 min at 37°C. Nuclei were stained with DAPI for 15 min at room temperature. Images were captured using the EVOS imaging system.

### 2.7 Indirect ELISA

The BVDV E2 antigen was diluted in carbonate buffer (CBS) to a concentration of 0.25 μg/mL, and 100 μL of this solution was added to each well of 96-well plates. The plates were incubated overnight at 4°C. The following day, the plates were washed three times with PBST (0.05% Tween in 0.01 M PBS) and blocked with 5% skimmed milk in PBST for 1 h at 37°C. After three additional washes with PBST, the purified monoclonal antibodies 3E6, 2D5, and 5B9 (2 mg/mL) were diluted starting at 1:500 and then serially diluted 2-fold. A total of 100 μL of each dilution was added to the wells and incubated at 37°C for 1 hour. The BVDV E2 immunized serum was used as the positive control and the non-immunized mice serum served as the negative control. After further washing, 100 μL of HRP-conjugated goat anti-mouse IgG, diluted 1:5,000 in PBST, was added to the plates and incubated for 1 h at 37°C. Following additional washes, 100 μL of tetramethylbenzidine (TMB) substrate solution was added to each well, and the plates were incubated at room temperature for 10 min, The reaction was stopped by adding 100 μL of 2 M H_2_SO_4_ per well. The results were measured at an optical density of 450 nm (OD_450_) using a microplate reader.

### 2.8 Virus neutralization test

Serum samples were tested for BVDV neutralizing antibodies following the guidelines of the World Organization for Animal Health (WOAH) Manual of Diagnostic Tests and Vaccines for Terrestrial Animals. Briefly, the test sera are heat-inactivated for 30 minutes at 56°C, then each serum sample was serially diluted two-fold, starting from a 1/4 dilution, and mixed separately with BVDV-1 (cytopathic NADL strain) or BVDV-2 (noncytopathic HLJ-10 strain) at 100 TCID_50_ per well. And the mixture was added to a 96-well microplate (100 µL per well, with four replicates for each dilution) and incubated at 37°C for 1 h. Subsequently, 1.50 × 10^4^ MDBK cells were added to each well, and the plate was incubated at 37°C for five days. Positive/negative sera control, virus infected/uninfected control were included in each test. The cytopathic strain infected samples were examined microscopically for cytopathic effect, and non-cytopathic strain infected samples were fixed and strained by immune-peroxidase staining using BVDV-1&2 mAb E2 gp53 IgG2b isotype. The neutralizing titer for each serum, calculated using the Reed-Muench method, was the dilution at which the virus was neutralized in 50% of the wells. A sample showed no neutralization at the dilution of 1/4 was considered negative, otherwise, it was considered positive. For serum samples, if the neutralizing antibody for either type 1 or type 2 was determined to be positive, the serum was regarded as a positive sample. Conversely, if both type 1 and type 2 neutralizing antibodies were determined to be negative, the serum is considered a negative sample.

### 2.9 Serum panel

Between April 2021 and December 2023, a total of 234 serum samples were collected from four different farms located in Heilongjiang Province (126 samples) and Inner Mongolia Autonomous Region (108 samples). These samples were previously determined to be positive or negative against the BVDV-1 and BVDV-2 strain through the VNT. The collected samples were then used to establish the cut-off value for the E2 cELISA by comparing the results of the cELISA to the VNT data.

To evaluate the ability of the E2 cELISA to detect immune sera, a total of 160 vaccinated serum samples were collected in March 2023 from three different farms in Heilongjiang Province. To evaluate the concordance of the developed cELISA with VNT, 190 clinical serum samples were collected in 2023 from eight cattle farms in Heilongjiang Province, Xinjiang Uygur Autonomous Region, Inner Mongolia Autonomous Region, and Henan Province. Among these, 78 samples were from five farms experiencing a BVDV outbreak, while 112 samples were from three cattle farms without BVDV infection. All serum samples were first inactivated at 56°C for 30 min.

### 2.10 Development of the cELISA

To assess the competitive ability of the mAbs, five positive sera and five negative sera were used. The 96-well ELISA plates were coated with the rpE2 overnight at 4°C. After washing the plates three times with PBST, they were blocked with 5% skimmed milk in PBST for 1 h at 37°C. A testing mixture containing 50 μL of serum samples and 50 μL of mAb were added to each well and incubated for 60 min at 37°C, followed by three-times’ washing with PBST. Next, 100 μL of HRP-conjugated goat anti-mouse IgG, diluted 1:5000 in PBST, was added to the plates and incubated for 1 h at 37°C. After additional washing, TMB substrate was added for color development, and the reaction was stopped with 2 M H_2_SO_4_. The results were measured at OD_450_ using a microplate reader. The percent inhibition (PI) was calculated using the following formula:


$$PI\;\left(\%\right)\;=\;\left[\frac{\left(mean\;OD\;of\;negative\;control\;-\;OD\;of\;sample\right)}{mean\;OD\;of\;negative\;control}\right]\;\times \;100\;\left(\%\right)$$

The high-purity mAb 3E6 was conjugated with HRP using EZ-Link^™^ Plus Activated Peroxidase (Thermo Scientific, NJ, USA) according to the manufacturer’s instruction. The HRP-conjugated 3E6 mAb was dialyzed against PBS (pH 7.4) at 4°C for 5 h, with the buffer replaced, and continued dialysis overnight at 4°C. Proclin 300 was then added to a final concentration of 0.1%, mixed with glycerol in a 1:1 ratio, aliquoted into 1.0 mL per tube, and stored at -20°C protected from light.

To optimize the cELISA protocol, a range of rpE2 proteins concentrations from 0.0625 μg/mL to 2 μg/mL was tested for coating. The dilution ratios of the HRP-conjugated 3E6 mAb were varied between 1:50 and 1:800. Optimal conditions were determined by achieving OD_450_ values close to 1.0 in an iELISA, using checkerboard titration. Three BVDV antibody-negative and positive sera were then tested at dilutions ranging from 1:1 to 1:64, and the optimal serum dilution was selected based on the smallest ratio of OD_450_ values between positive and negative sera (P/N). The incubation time for the sera and HRP-conjugated mAb 3E6 were evaluated at intervals of 30, 60, 90, and 120 mins, and the colorimetric reaction time after TMB addition was assessed at 5, 10, 15, and 20 mins. The optimal conditions were chosen based on the smallest P/N ratio, with the final procedure providing the highest differentiation between the positive and negative reference sera.

### 2.11 Validation of the BVDV E2 cELISA

Forty serum samples from cattle previously infected with or vaccinated against various bovine viruses, including BVDV, BCoV, BHV-1, BRV, AKAV, LSDV, and BLV, and the serum samples of CSFV were provided by Dr. Huaji Qiu at Harbin Veterinary Research Institute, were collected to assess the specificity of the BVDV E2 cELISA. The repeatability of the cELISA was evaluated using eight serum samples-four negative and four positive samples for BVDV. Consistency was measured by calculating the coefficient of variation (CV), defined as (standard deviation SD/mean) × 100%. Intra-assay CV was determined by analyzing each sample in three replicates on one plate, while inter-assay CV was calculated from three separate measurements of each serum sample.

### 2.12 Immunization of calve with BVDV-1 and BVDV-2

MDBK cells were seeded in T225 cell flasks at a density of 5 × 10^7^ cells and infected with BVDV-1a and BVDV-2a at an MOI of 1, respectively. At 96 hours post-infection (h.p.i.), virus was harvested from both cells and supernatant using three freeze-thaw cycles. The lysates were then collected and centrifuged at 3,000 rpm for 15 minutes to remove cell debris. The resulting supernatant was further centrifuged at 10,000 rpm for 30 minutes, followed by a final centrifugation at 29,300 rpm for 2 h. The supernatant was discarded, and the pellet was resuspended in 1 mL per tube of DMEM and incubated overnight at 4°C. Then virus titers of BVDV-1 and BVDV-2 were determined by TCID_50_ assay.

BVDV-1 and BVDV-2 were inactivated with binary ethyleneimine (BEI, final concentration of 4 mM) at 30°C for 24 h. Inactivated BVDV-1 or BVDV-2 was mixed with adjuvant ISA 15A VG at a ratio of 85:15 (v/v) respectively. A total of eight BVDV-negative calves aged 3-6 months were divided into the BVDV-1 immunization group (n=3), BVDV-2 immunization group (n=3) and control group (n=2). Each calf of immunization groups received intramuscular immunization in the neck with 2 mL of inactivated BVDV-1/BVDV-2 with viral titer 10^8^ TCID_50_/mL. Boosted immunization on week 2 and week 4 after primary immunization. Blood samples were collected from all immunized cattle once every two weeks following the injections.

### 2.13 AlphaFold 3 assay

The mAb 3E6 was sequenced by Novoprotein Scientific Inc and then the crystal structural models of BVDV E2 protein and mAb 3E6 were constructed by AlphaFold 3. The complex structures of the BVDV E2 with the mAb 3E6 were determined by AlphaFold 3 and analyzed by Schrödinger (28).

### 2.14 Statistical analysis

ROC curve analysis was conducted using 99 sera from the BVDV antibody positive animals and 135 sera from the BVDV antibody negative animals to determine the cut-off value, sensitivity, and specificity of the BVDV E2 cELISA. Statistical analysis and data visualization were carried out using GraphPad Prism software (version 9.4.1; GraphPad Software, Inc., La Jolla, CA). The degree of agreement (kappa value) between the cELISA and VNT was calculated using Microsoft Excel. A higher kappa value indicates better consistency between the two methods, a kappa value between 0 and 0.40 suggests poor consistency, and a kappa value over 0.40 indicates high consistency.

## 3 Results

### 3.1 Expression of BVDV E2 protein and identification of its immunogenicity

**Figure 1:**
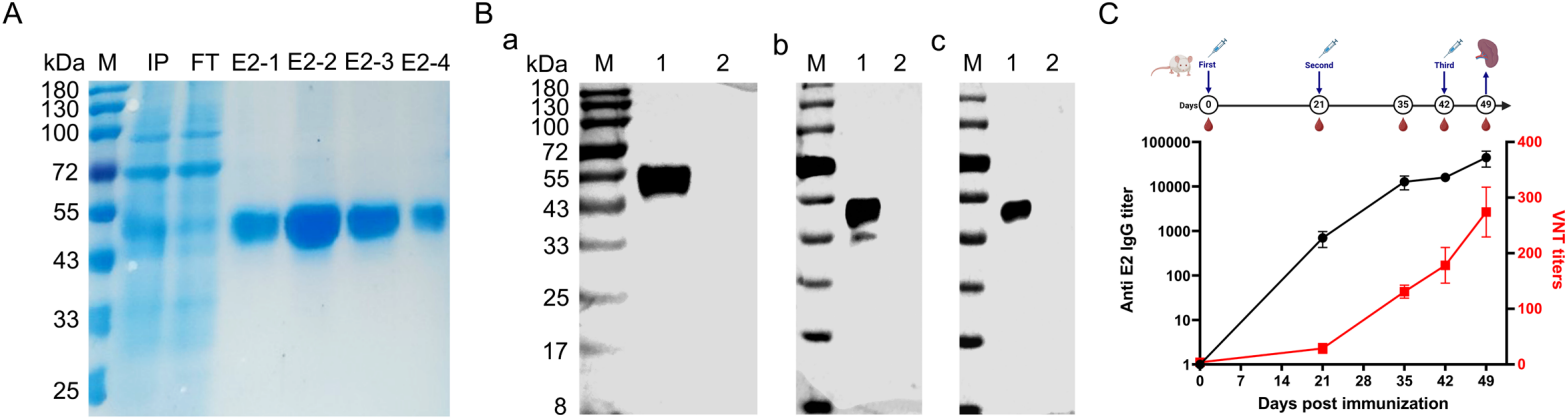
Expression of BVDV E2 protein and identification of its immunogenicity. **(A)** SDS-PAGE analysis of rpE2, M, protein marker; IP, culture medium supernatant after transfection; FT, flow-through after incubation with Ni^2+^ NTA affinity column; E2-1, E2-2, E2-3, and E2-4, purified BVDV E2 proteins. **(B)** Western blotting analysis of the purified rpE2 with various antibodies. (a) Incubated with His-tag mouse mAb; (b) Incubated with BVDV antiserum; (c) Incubated with bovine viral diarrhea virus type 1&2 (BVDV-1&2) mAb E2 gp53 IgG2b isotype. M, protein marker; lane 1, purified BVDV E2 protein with His tag; lane 2, cell culture medium. **(C)** ELISA and virus neutralization test analysis of anti-BVDV E2 serum.

The amplified BVDV E2 gene (Δ30 amino acid) was cloned into the pCAGGS-6 × His vector with the IL10 signal peptide and subsequently transfected into Expi293F cells. Following the transfection, the rpE2 protein was expressed in a soluble form in the supernatant and subsequently purified using a Ni^2+^ NTA affinity column. SDS-PAGE analysis revealed that a predominant band corresponding to BVDV E2 at approximately 55 kDa in lane IP, with minimal stray bands observed after purification (**Figure 1A**). Western blot analysis further confirmed that a single protein band corresponding to BVDV E2 was specifically recognized by the His-tag mouse mAb (**Figure 1B**-a), BVDV antiserum (**Figure 1B**-b), and BVDV type 1&2 (BVDV-1&2) mAb E2 gp53 IgG2b isotype (**Figure 1B**-c).

To assess the immunogenicity of the rpE2, BALB/c mice were immunized with the rpE2, and serum was collected on days 0, 21, 35, 42, and 49. As shown in **Figure 1C**, the immunization successfully induced both IgG antibodies and virus-neutralizing antibodies. The anti-BVDV E2 IgG titer reached approximately 1:100,000, and the virus-neutralizing test titer was around 1:300 (**Figure 1C**). These results demonstrated that the rpE2 is highly immunogenic, indicating its potential as a candidate for further development in diagnostic applications.

### 3.2 Generation of mAbs against BVDV E2

**Figure 2:**
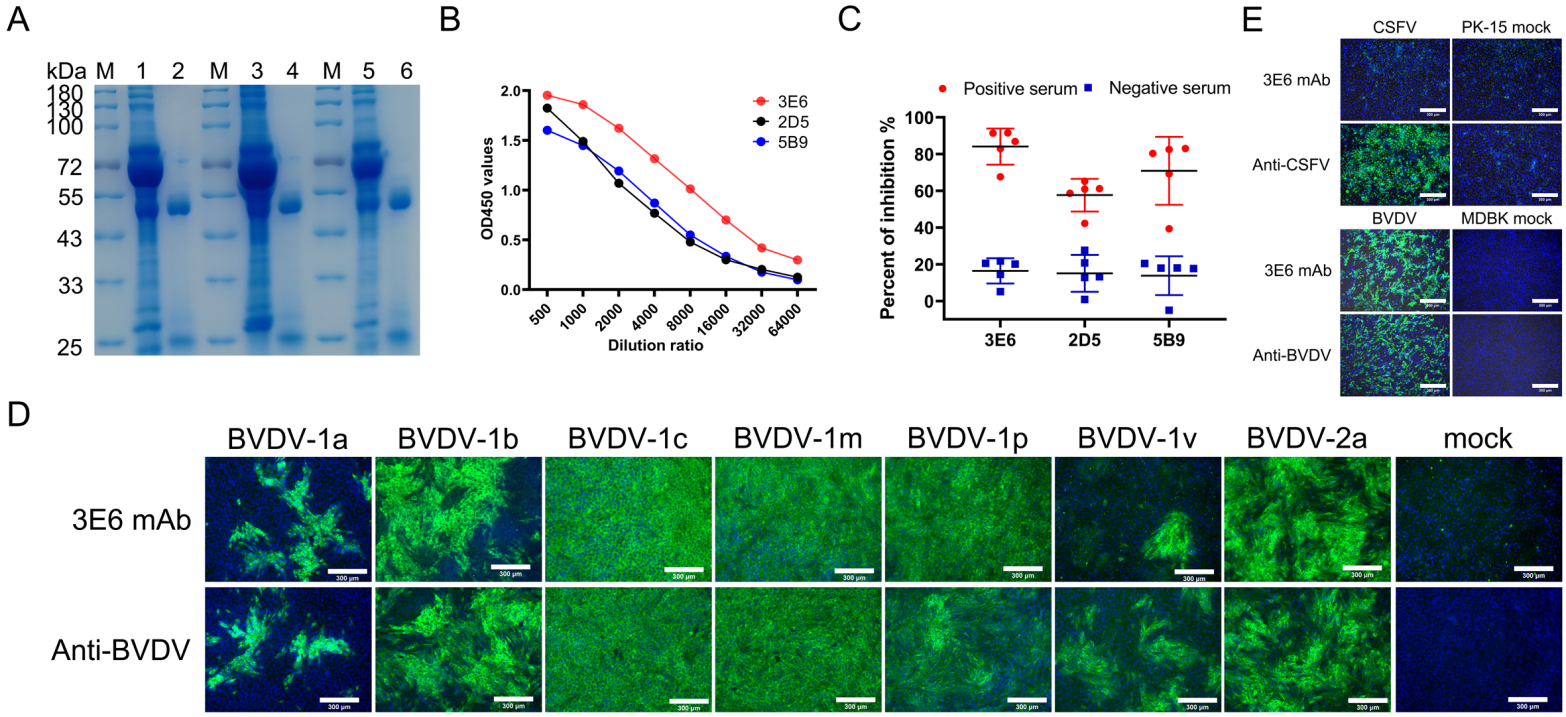
Characterization of mAbs 3E6, 2D5, and 5B9. **(A)** SDS-PAGE analysis of purified BVDV E2 mAbs 3E6, 2D5, and 5B9. M, protein marker; lane1, mAb 3E6 pre-purification; lane 2, mAb 3E6 post-purification; lane 3, mAb 2D5 pre-purification; lane 4, mAb 2D5 post-purification; lane 5, mAb 5B9 pre-purification; lane 6, mAb 5B9 post-purification. **(B)** Comparison of mAb reactivity to rpE2 by iELISA. **(C)** Evaluation of the ability of mAbs to differentiate positive sera and negative sera using cELISA. Each symbol shape represents the PI of serum for each respective mAb. Five positive BVDV sera (Red) and five negative sera (Blue) were tested, and the average PI for positive and negative sera was recorded for each mAb. **(D)** Evaluation of the broadly specificity of mAb 3E6 for various BVDV genotypes by IFA. Cells infected with BVDV-1a, BVDV-1b, BVDV-1c, BVDV-1m, BVDV-1p, BVDV-1v, and BVDV-2a were immunostained with mAb 3E6 at 60 h.p.i., while uninfected cells served as negative controls. Cells were infected BVDV-1a and BVDV-1v at MOI of 0.5, or BVDV-1b and BVDV 2a at MOI of 1, BVDV 1c, BVDV 1m, and BVDV-1p at MOI of 2. Scale bar, 300 μm. **(E)** Assessment of the specificity of mAb 3E6 against CSFV and BVDV by IFA. PK-15 cells infected with CSFV and MDBK cells infected with BVDV were immuno-stained with 3E6 mAb, anti-CSFV serum, and a commercial anti-BVDV mAb, with uninfected cells as negative controls. Scale bar, 300 μm.

MAbs were prepared using hybridoma technology as reported previously (29). Three clones that produced antibodies specifically targeting the E2 protein were obtained, and were designated as 3E6, 2D5, and 5B9. The isotypes of mAbs 3E6 and 2D5 were identified as IgG1 with the kappa light chain, while mAb 5B9 was classified as IgG1 with the lambda light chain (**Table 1**). Three mAbs were purified from the ascites fluid of mice injected with hybridoma cells and their purity were subsequently analyzed using SDS-PAGE. The heavy and light chains of the mAbs were detected at approximately 53 kDa and 25 kDa, respectively (**Figure 2A**). Furthermore, the binding abilities of the three mAbs were further assessed using iELISA. Initially, the mAbs were set at a concentration of 2 mg/mL and then serially diluted in a 2-fold ratio starting from 1:500. Analysis of the interactions between the serially diluted mAbs and rpE2 showed that mAb 3E6 exhibited the highest titer and demonstrated exceptional immunoreactivity against rpE2 (**Figure 2B**).

**Table 1:** Identification of isotypes of E2 mAbs.

	Monoclonal Antibodies		
	2D5	5B9	3E6
Ig subclass	IgG1	IgG1	IgG1
Light chain type	Kappa	Lambda	Kappa

To evaluate the competitive binding capability of the three mAbs to rpE2, cELISA was conducted using five positive sera and five negative sera. The results indicated that all five positive BVDV sera effectively interfered with mAb 3E6 by more than 67.68%, with a mean PI value of 84.11%, while the mean PI values for the five negative sera remained at 16.41%. For mAbs 2D5 and 5B9, the mean PI value for the five positive BVDV sera were 57.67% and 70.91%, respectively. Thus, mAb 3E6 demonstrated the strongest effective competitive activity among the three mAbs (**Figure 2C**).

Considering BVDV is divided into various genotypes, we evaluated the broad reactivity of mAb 3E6 through IFA. The results showed that mAb 3E6 specifically interacted with various genotypes of BVDV (**Figure 2D**), demonstrating its characteristics of pan-genotypic. Since BVDV belongs to the *Pestivirus* genus within the *Flaviviridae* family, which also includes CSFV. Notably, the E2 protein of BVDV shows a high sequence homology with the E2 protein of CSFV. We further evaluated the specificity of the mAb 3E6 by testing its recognition of both CSFV and BVDV using IFA. The results confirmed that the mAb 3E6 specifically recognizes BVDV and does not cross-react with CSFV (**Figure 2E**). Therefore, given the excellent reactivity, specificity, and competitive activity of mAb 3E6, it held the potential as a detection antibody for subsequent cELISA development.

### 3.3 Prediction of antigenic epitopes

**Figure 3:**
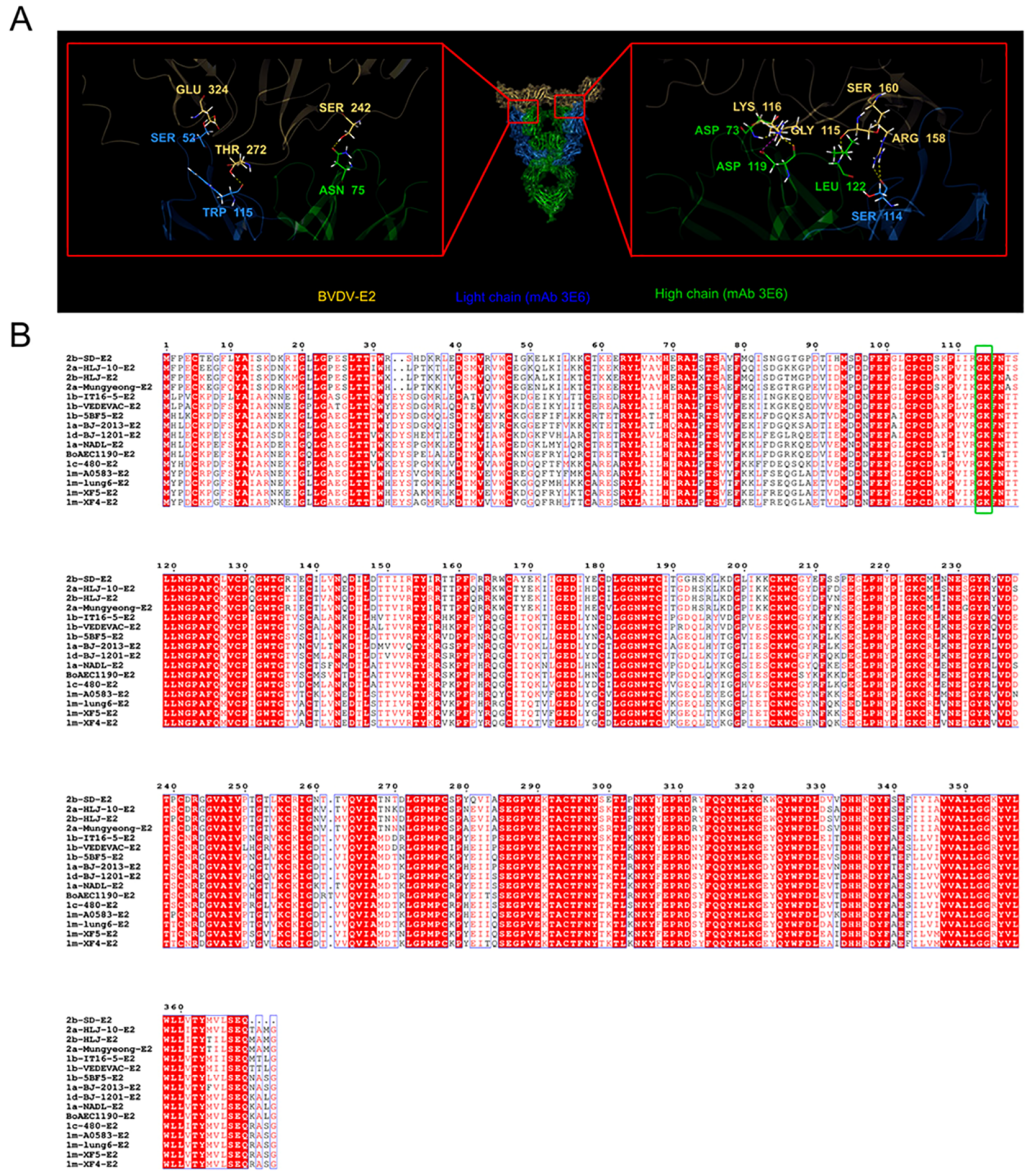
AlphaFold 3 predicted the key sites for the interaction between BVDV E2 and mAb 3E6. **(A)** AlphaFold 3 prediction results of the key sites of BVDV E2 protein (yellow) binding to mAb 3E6 (blue: light chain; green: high chain). **(B)** Comparison of E2 sequences of different BVDV strains. The green box shows the conserved binding sites Gly115 and Lys116 of BVDV E2 and mAb 3E6.

E2 is an envelope protein of BVDV and serves as a basis for classifying different BVDV genotypes (15). Our results showed that the mAb 3E6 could specifically recognize various BVDV genotypes. We hypothesized that the mAb 3E6 specifically recognizes a conserved region of BVDV E2. To investigate this, we sequenced the mAb 3E6 antibody, and then predicted the interaction sites between E2 and the mAb 3E6 using AlphaFold 3 (**Figure 3**). The analysis revealed that MAb 3E6 predominantly interacted with the domain B of the BVDV E2 protein, where amino acids Asp73 and Asp119 of the mAb 3E6 light chain form hydrogen bonds with amino acids Gly115 and Gly116 of BVDV E2, respectively (**Figure 3A**). Notably, these interaction sites were highly conserved across different BVDV genotypes (**Figure 3B**), suggesting that mAb 3E6 has the potential to broadly recognize various forms of the BVDV E2 proteins. These finding underscored the utility of mAb 3E6 in diagnostics and potential therapeutic applications against BVDV.

### 3.4 Establishment and optimization of cELISA based on mAb 3E6

**Figure 4:**
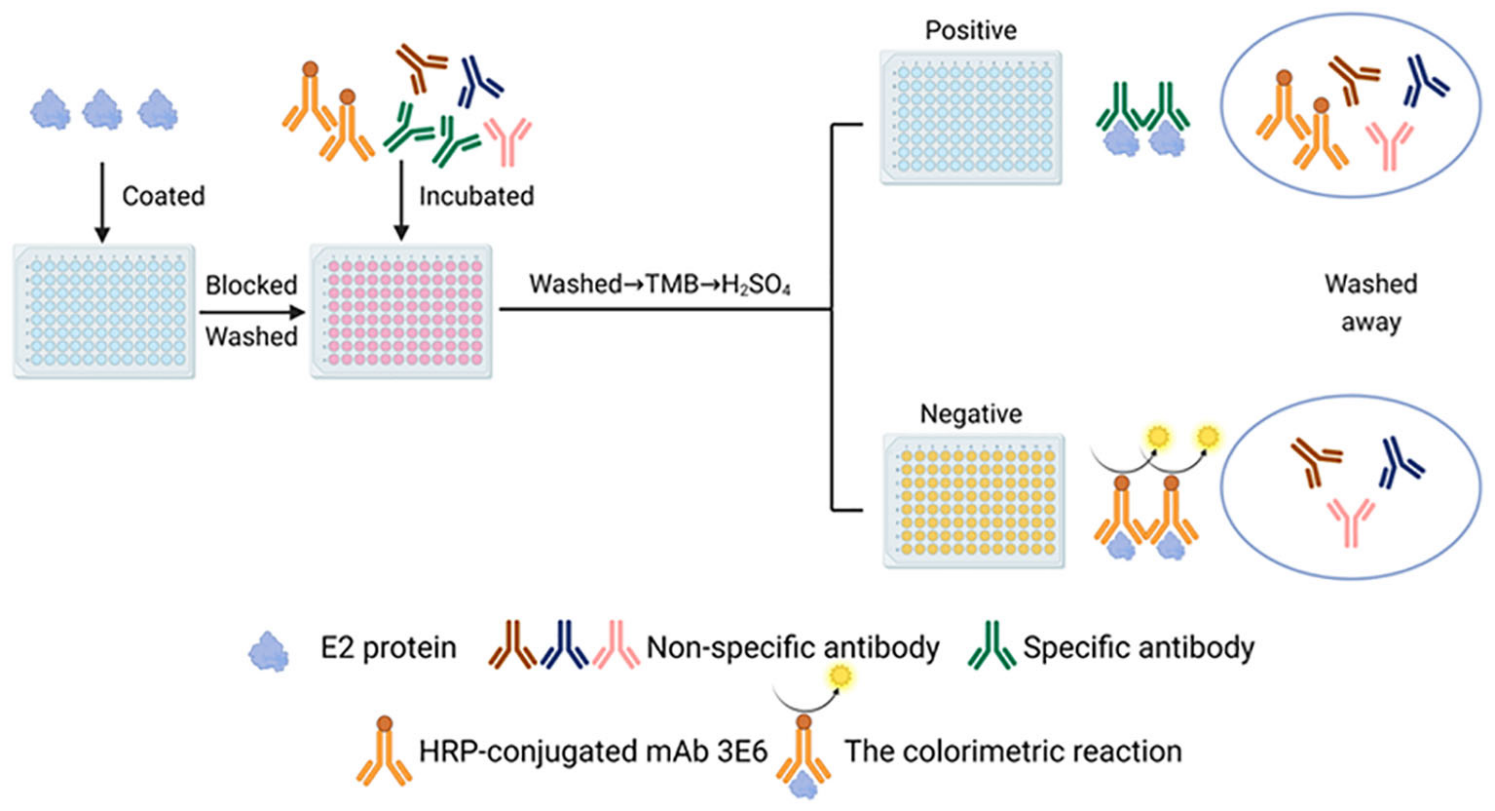
Schematic diagram of the BVDV E2 antibody cELISA. The Figure was generated using BioRender (https://www.biorender.com/library).

**Figure 5:**
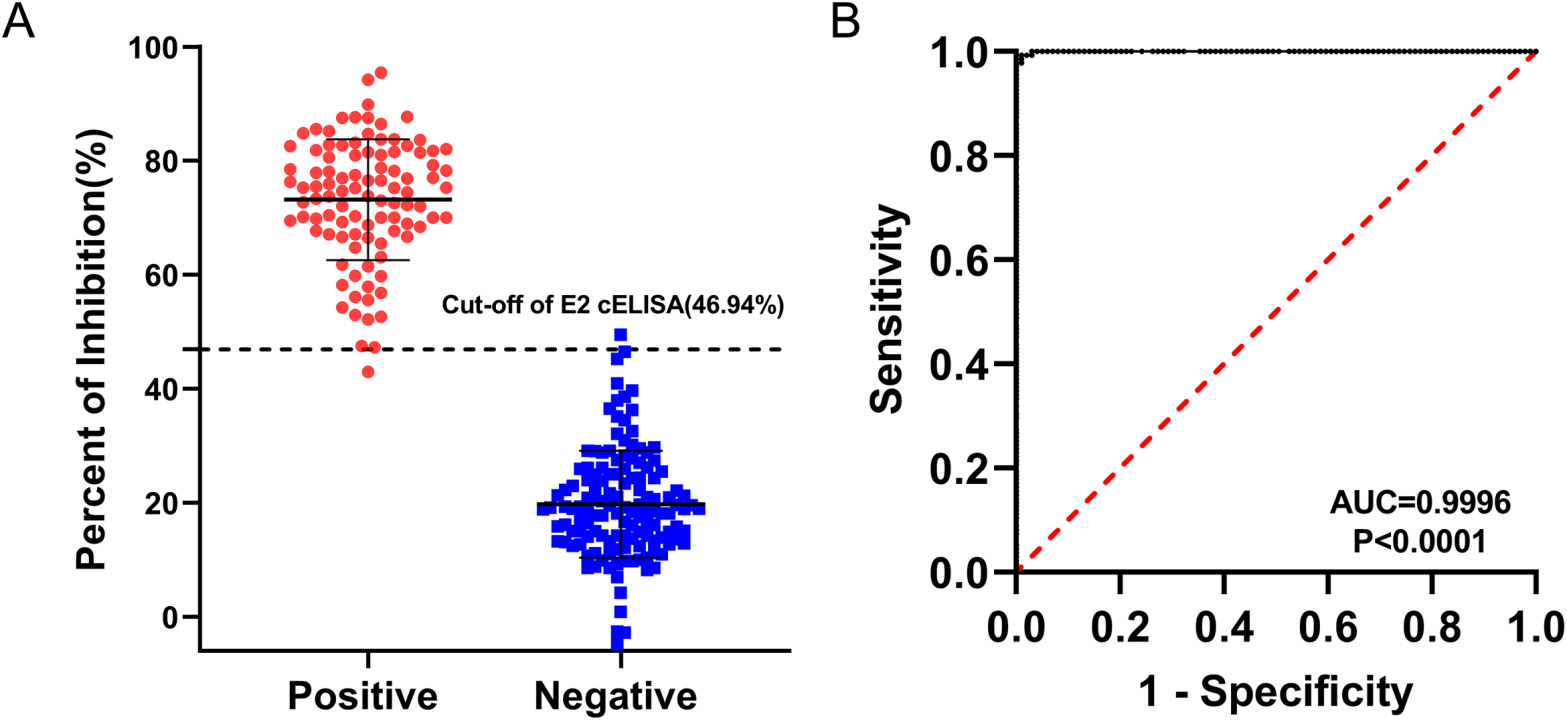
ROC analysis for the BVDV E2 cELISA. The assay was conducted using BVDV-negative sera (n = 135) and BVDV-positive sera (n = 99). **(A)** Interactive dot plot diagram displaying the PI values of sera while the cut-off value was set to 46.94%. **(B)** ROC analysis of cELISA results while the AUC of the test was 0.9996.

Based on the checkerboard titration results, the optimal dilution for the 3E6 mAb was determined to be 1:200, and the optimal coating concentration for the BVDV E2 protein was established as 0.25 μg/mL (**Supplementary Table S1**). Accordingly, the optimal dilution for the sera was found to be 1:2 (**Supplementary Table S2**). The checkerboard method also determined that the optimal incubation time for sera and mAb was 1 h, while the optimal colorimetric reaction time was 10 minutes. These conditions established the best reaction parameters for the BVDV E2 cELISA (**Table 2**), and the detection process was performed for further evaluation (**Figure 4**). To determine the cut-off value, as well as sensitivity and specificity of the BVDV E2 cELISA, a set of 135 negative sera and 99 positive sera were used. The percentage of inhibition values for each sample were calculated and plotted in an interactive scatter plot (**Figure 5A**). ROC analysis was conducted to identify the optimal cut-off value for diagnostic sensitivity and specificity (**Figure 5B**). Based on the ROC analysis, the area under the curve (AUC) was determined to be 0.9996 (95% confidence interval: 0.9989 to 1.00). Furthermore, when the cutoff value of the developed cELISA was set at 46.94%, the diagnostic sensitivity and specificity were 99.26% (95% confidence interval: 0.9592 to 0.9996) and 98.99% (95% confidence interval: 0.9450 to 0.9995), respectively. Thus, the cut-off value for the PI was ultimately set at 46.94%. Consequently, serum samples with a PI value < 46.94% were considered negative, while serum samples with a PI value ≥ 46.94% were classified as positive. Notably, among the 135 analyzed negative sera, only one was erroneously identified as false positive with PI values of 49.51%. Among the 99 positive sera, only 1 was classified as false negative, with a PI value of 42.97%.

**Table 2:** Optimized conditions of the BVDV E2 cELISA.

Optimized dilutions and reaction conditions	E2 cELISA
Coating condition	0.25 μg/well in CBS
	4°C, 16 h
Blocking condition	5% skimmed milk
	37°C, 1 h
Untested sample and the HRP-conjugated mAb 3E6	1:2 and 1:200, respectively
	37°C, 1 h
Chromogenic substrate	100 μL
	RT, 10 mins

### 3.5 Specificity and sensitivity of the BVDV E2 cELISA

**Figure 6:**
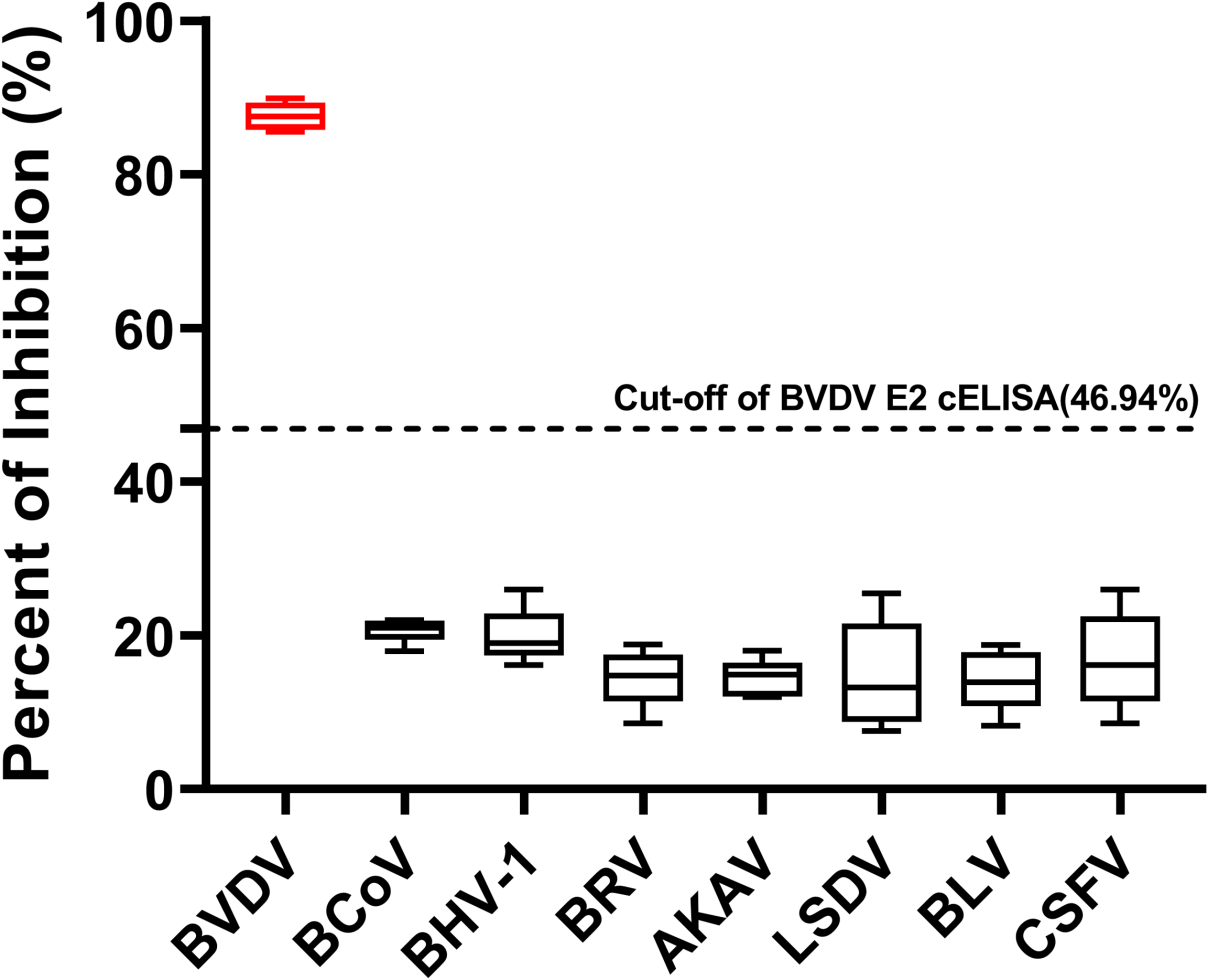
Specificity of the BVDV E2 cELISA. Evaluation of the cELISA for detecting antibodies against seven bovine viruses, including BVDV, BCoV, BHV-1, BRV, AKAV, LSDV, and BLV, and pestivirus CSFV. The horizontal dotted line represents the cutoff value.

To confirm the specificity of the cELISA, we tested positive sera specifically against six bovine viruses including BCoV, BHV-1, BRV, AKAV, LSDV, and BLV, as well as pestivirus CSFV, alongside BVDV positive sera. The PI values for the sera against the six bovine viruses were all below 46.94%, whereas the PI values for the BVDV positive sera exceeded 46.94%. These results indicated that the cELISA exclusively detected antibodies against BVDV and did not cross-react with antibodies against other bovine viruses and CSFV (**Figure 6**).

**Figure 7:**
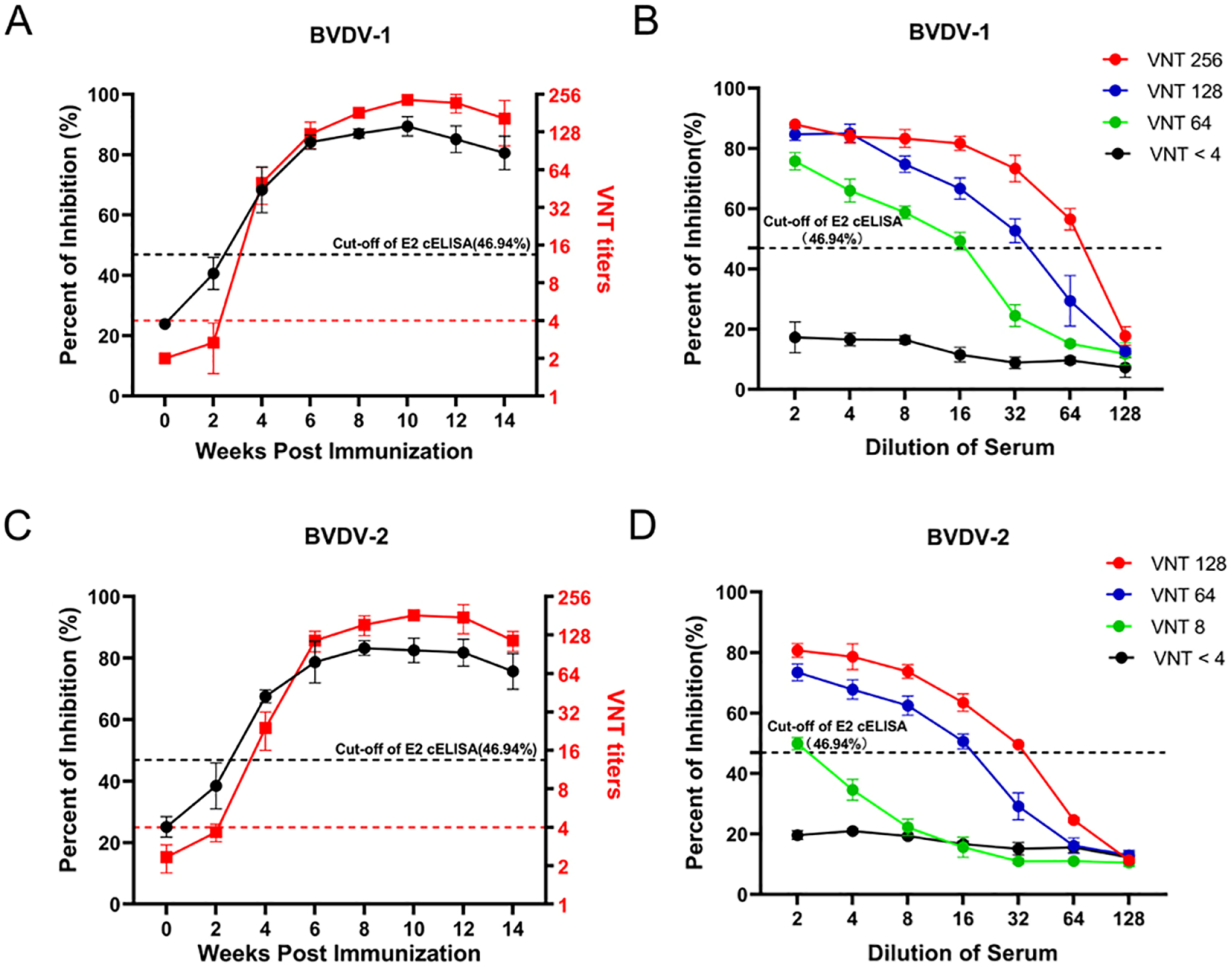
Detection of the antibody after BVDV immunization in calves using the BVDV E2 cELISA and the VNT. **(A)** Comparison of the ability of BVDV E2 cELISA and VNT methods in detecting the antibody dynamics after BVDV-1 immunization. The black horizontal dotted line represents the cut-off value of BVDV E2 cELISA and the red horizontal dotted line represents the cut-off value of VNT titers. **(B)** Detection of the serial dilutions of serum samples against BVDV-1 with different neutralizing titers by the E2 cELISA. The black horizontal dotted line represents the cut-off value. **(C)** Comparison of the ability of BVDV E2 cELISA and VNT methods in detecting the antibody dynamics after BVDV-2 immunization. The black horizontal dotted line represents the cut-off value of BVDV E2 cELISA and the red horizontal dotted line represents the cut-off value of VNT titers. **(D)** Detection of the serial dilutions of serum samples against BVDV-2 with different neutralizing titers by BVDV E2 cELISA. The black horizontal dotted line represents the cut-off value.

Furthermore, to evaluate the sensitivity of the developed cELISA, we immunized 6 calves with inactivated BVDV-1 (3) and BVDV-2 (3), respectively. Then assessed serum samples from BVDV-immunized calves at different time points post-immunization using both the BVDV E2 cELISA and the VNT. As shown in **Figure 7A**, the seroconversions of BVDV-1 immunized calves were firstly detected at the 4th week after immunization by both the VNT and the E2 cELISA, and the antibody dynamics showed a similar trend with the detection of the two methods. Moreover, four sera with different neutralizing antibody titers (VNT < 4, VNT 64, VNT 128, VNT 256) against BVDV-1 were selected to detected by the cELISA with 2-fold dilution. It was observed that the PI value of the sera gradually decreased with the dilutions, and the maximum dilutions of the VNT 64, VNT 128 and VNT 256 judged to be positive in the cELISA were 16, 32 and 64, respectively (**Figure 7B**). Similarly, the seroconversions of BVDV-2 immunized calves were also firstly detected at the 4^th^ week after immunization by both VNT and the E2 cELISA (**Figure 7C**), and the PI value of the sera with different neutralizing titers against BVDV-2 decreased regularly with dilutions (**Figure 7D**). These results indicate that cELISA could sensitively monitor the dynamics of antibodies against BVDV-1 and BVDV-2 in serum, and that the PI value of the cELISA corresponds well to the neutralizing antibody titer.

### 3.6 Repeatability and reproducibility of the BVDV E2 cELISA

Four BVDV positive sera and four BVDV negative sera were assessed using the developed BVDV E2 cELISA, and triplicate runs were performed on one plate as well as three independent measurements for each serum sample. This evaluation was conducted to assess the repeatability and reproducibility of the cELISA. As shown in **Table 3**, the intra-assay coefficient of variation (CV) for the PI ranged from 0.11% to 3.23%, while the inter-assay CV ranged from 0.95% to 4.38%. These results indicated remarkable repeatability and reproducibility of the BVDV E2 cELISA.

**Table 3:** Repeatability of the BVDV E2 cELISA.

Samples	Repeatability (Intra-assay)			Reproducibility (Inter-assay)		
	Mean PI (%)	SD	CV (%)	Mean PI (%)	SD	CV (%)
1	94.71	0.47	0.49	94.44	1.04	1.10
2	87.04	1.67	1.92	88.02	1.70	1.93
3	69.79	0.08	0.11	70.18	0.75	1.07
4	82.86	0.86	1.03	82.05	0.78	0.95
5	4.80	0.15	3.23	4.74	0.21	4.38
6	22.82	0.55	2.41	22.17	0.67	3.02
7	38.47	0.25	0.65	38.27	0.45	1.17
8	10.95	0.16	1.47	10.74	0.45	4.17

### 3.7 Clinical performance assessing of the BVDV E2 cELISA

A total of 350 clinical serum samples, including 160 from vaccinated animals and 190 from clinical cases, were tested for BVDV using both the developed cELISA and the VNT. The developed cELISA demonstrated a 100% coincidence rate (160/160) with the VNT for vaccinated sera and a 95.8% coincidence rate (182/190) for clinical sera (**Table 4**). Statistical analysis revealed no significant difference between the cELISA and the VNT with the kappa values of 1 and 0.908, respectively (**Table 4**) (all kappa values were > 0.4). These results demonstrated that the BVDV E2 cELISA had a high level of consistency with the VNT, highlighting its strong potential for clinical detection.

**Table 4:** Comparison of the BVDV E2 cELISA with virus neutralization test (VNT).

Serum			VNT		Agreement (%)^a^	Kappa value^b^
	cELISA	No	Positive	Negative		
Vaccinated sera	Positive	156(A)	156(B)	0	100	1
	Negative	4(C)	0	4(D)		
Clinical sera	Positive	66(A)	64(B)	2	95.8%	0.908
	Negative	124(C)	6	118(D)		

a Agreement (%) =(B+D)/(A+C).

b The kappa value > 0.4 was regarded as significant difference.

## 4 Discussion

Bovine viral diarrhea (BVD) is an acute, febrile, contagious disease caused by the BVDV. This disease is widely prevalent in many countries around the world. In addition, BVDV often leads to mixed infections with multiple pathogens, thus posing a significant threat to the global ruminant farming industry (3, 4, 30). BVDV can contaminate biological products such as vaccines, interferons, and frozen semen through serum, significantly threatening the safety and efficacy of these products (31–33). Therefore, establishing a rapid and efficient diagnostic method for BVDV is particularly important. Currently, the diagnostic methods for BVDV primarily include: 1. Etiological detection methods (e.g., virus isolation) (21); 2. Serological methods [e.g., VNT (21), ELISA (24, 25), IFA (34), IHC (35)]; 3. Molecular methods [e.g., RT-PCR (36), Real-Time PCR (37), Digital Droplet PCR (38), Loop-Mediated Isothermal Amplification (39), Recombinase Polymerase Amplification (40), and CRISPR-Cas systems (41)]; 4. Biosensor methods (42).For serological detection methods, VNT and ELISA are primarily utilized. Compared to the VNT, ELISA is simpler, faster, and capable of testing a large number of samples. Currently, there are several types of commonly used methods for ELISA, including indirect, blocking, and competitive ELISA that target antigens such as E2, NS3, and E^rns^. Among these, the E2 protein of BVDV is the main target of the immune response and plays a crucial role in virus neutralization.

In this study, unlike previous studies that expressed partial fragments of E2 (27), we expressed the BVDV E2 protein, which only lacked transmembrane region, avoid missing important antigen epitopes for selection of highly effective and broad-spectrum antibodies. Since E2 protein is an envelope glycoprotein of BVDV, and the proteins expressed in prokaryotic systems lack post-translational glycosylation modifications (43), so we expressed E2 in a eukaryotic system to ensure that it adopts the correct conformation. In order to improve the expression level of E2 protein, we selected the highly efficient suspension cell expression system-Expi293F, and to enhance the efficiency of protein purification, the E2 protein with IL10 signal peptide sequence was expressed in large quantities in the cell supernatant in the secretion form. As shown in the **Figures 1A, B**, the recombinant E2 protein, purified from Expi293F cells, yielded a predominant band at approximately 55 kDa, confirming its correct expression and allowing for subsequent analyses. The strong immune response observed in BALB/c mice, with anti-BVDV E2 IgG titers reaching approximately 1:100,000 and virus-neutralizing antibody titers around 1:300 (**Figure 1C**), highlights the protein’s effectiveness as an immunogen. This indicates that the E2 protein has good properties and has the potential to establish a method.

The generation of three mAbs against the BVDV E2 protein, particularly mAb 3E6, further supports our goal of establishing a reliable diagnostic tool (**Figures 2A, B**). The competitive ELISA results revealed that mAb 3E6 exhibited the highest immunoreactivity and competitive binding activity, significantly differentiation the positive and negative sera (**Figure 2C**). This suggested that mAb 3E6 is not only effective for detecting BVDV E2 but also has the potential for broader applications in diagnostics and research. Our findings indicate that mAb 3E6 can specifically recognize multiple genotypes of BVDV (**Figure 2D**). To further confirm the specificity of the cELISA, we tested positive sera specifically against CSFV and six other bovine viruses including BCoV, BHV-1, BRV, AKAV, LSDV, and BLV (**Figure 6**). For CSFV, the commercialized E2-based ELISA are unable to distinguish CSFV from BVDV infection, often resulting in false positives (44, 45). And there was serological cross-reactivity between specific antibodies against CSFV and BVDV in pigs (8, 46, 47). For six other bovine viruses, they often present as mixed infections in clinical samples, and they are the main respiratory, digestive, insect-borne and tumor-causing viruses currently prevalent in cattle. Like BVDV, BHV-1 is also a pathogen of bovine respiratory syndrome. It is also the pathogen of infectious pustular vaginitis and infectious balanoposthitis (48). BRV and BCoV are the main pathogens of neonatal calf diarrhea, which can cause serious economic losses (49). AKAV and LSDV are important insect-borne virus pathogens, which are transmitted by *Culicoides* and mosquitoes. AKAV can cause clinical symptoms such as abortion, premature birth, stillbirth, congenital malformation of the fetus and encephalomyelitis in animals, while LSDV can cause symptoms such as increased mortality, reduced hide quality, decreased milk production and slow weight gain in cattle, which seriously threaten the development of the cattle industry (50–52). BLV is a major cause of neoplastic disease in cattle and can cause economic losses (53). In our study, we validated that the established BVDV E2 cELISA exclusively detects antibodies against BVDV and does not have cross-reaction with antibodies against CSFV and six other bovine viruses.

The pan-genotype recognition of mAb 3E6 suggested that it targets a conserved epitope on the E2 protein. The prediction of interaction sites via AlphaFold 3 revealed that mAb 3E6 binds to the domain B of the E2 protein, further substantiating its potential as a diagnostic antibody (**Figure 3**). Since the E2 protein may also vary in different virus strains, if the cELISA cannot recognize the antibodies of various virus strain well, it may lead to missed detection (10). Therefore, we compared the BVDV E2 sequences of different genotypes and found that in the E2 and mAb 3E6 binding sites, Gly115 and Lys116 are highly conserved in different genotypes BVDV E2 (**Figure 3B**), which is consistent with the results of IFA that mAb 3E6 can recognize different BVDV-1 and BVDV-2 strains (**Figure 2D**). It provides a theoretical basis for determined the broadly detection range of the established cELISA and compensates for experimental biases that may be caused by the limited number of strains and serum samples. For the sites predicted by AlphaFold 3, we will further verify them in subsequent experiments.

By optimizing the cELISA blocking solution, antibody incubation time, and colorimetric reaction time, we established a convenient, time-saving, and economical BVDV E2 cELISA. BVDV not only infects cattle, but also pigs and other *Artiodactyls* (5–7). Unlike the cumbersome steps of E2 iELISA which requires the replacement of different secondary antibodies to detect different animal sera (54), the established BVDV E2 cELISA simplified the operation steps by labeling the monoclonal antibody with HRP (**Figure 4**). E2 is the most abundant surface structural protein of BVDV (55, 56). Due to its good immunogenicity, it can induce a strong humoral immune response and is the most important antigen component in the vaccine (57–59). Therefore, the method we established can detect the antibody dynamics upon animal immunization (**Figures 7A, C**). More importantly, the gold standard for vaccine immunity evaluation is the neutralizing antibody level, and the PI value of our method were highly correlated with the neutralizing antibody titer (**Figures 7B, D**). Thus, our method has the potential ability for detecting neutralizing antibody to evaluate whether the vaccine immunity is qualified. Of course, for animals in non-immune areas, this method can make accurate diagnosis of infected animals, evaluating the prevalence level of BVDV in the group. Therefore, the method we established had wide application for different hosts and had more diverse application scenarios, it posed extremely high value in antibody diagnosis and evaluation.

Additionally, the cELISA exhibited remarkable repeatability and reproducibility, with low coefficients of variation for both intra-assay and inter-assay evaluations (**Table 3**). These results are essential for ensuring consistent diagnostic outcomes across different testing environments, which is vital for clinical applications. Finally, the clinical performance assessment revealed a high coincidence rate with the VNT in antibody detection of vaccinated animals and clinical cases. The strong kappa values indicate substantial agreement between the two methods, suggesting that the cELISA can serve as a reliable alternative to traditional assays for BVDV detection (**Table 4**).

In conclusion, this study developed a BVDV E2 cELISA using a pan-genotypic mAb 3E6 recognized conserved epitope of BVDV. The established cELISA exhibited a high degree of specificity, sensitivity, and reproducibility. In clinical testing, the cELISA showed high agreement with the VNT. Therefore, the cELISA developed in this study is a valuable, simple, and reliable tool for serodiagnosis and antibody monitoring after. Future work should focus on validating the cELISA in different field settings and exploring its application in different animal species.

## Data Availability

The datasets presented in this study can be found in online repositories. The names of the repository/repositories and accession number(s) can be found in the article/**Supplementary Material**.

## Supplementary material

Supplementary Figure 1 Optimization of experimental method conditions for BVDV E2 cELISA. **(A)** Evaluation of blocking buffers (1% BSA, 5% skimmed milk, commercial blocking solution, and 2% gelatin) for optimization of BVDV E2 cELISA. **(B)** Evaluation of the incubation time of the sera and HRP-conjugated mAb 3E6 for BVDV E2 cELISA (30, 60, 90, and 120 mins). **(C)** Evaluation of colorimetric reaction times (5, 10, 15, and 20 mins) for TMB buffer used to optimize BVDV E2 cELISA.
