# Supplementary figures and images for: Development of a pan-genotypic monoclonal antibody-based competitive ELISA for the detection of antibodies against Bovine viral diarrhea virus
Source: Front Immunol. 2024 Nov 25;15:1504115. doi: 10.3389/fimmu.2024.1504115 (PMC11625775; doi:10.3389/fimmu.2024.1504115)

**Blocking Buffer**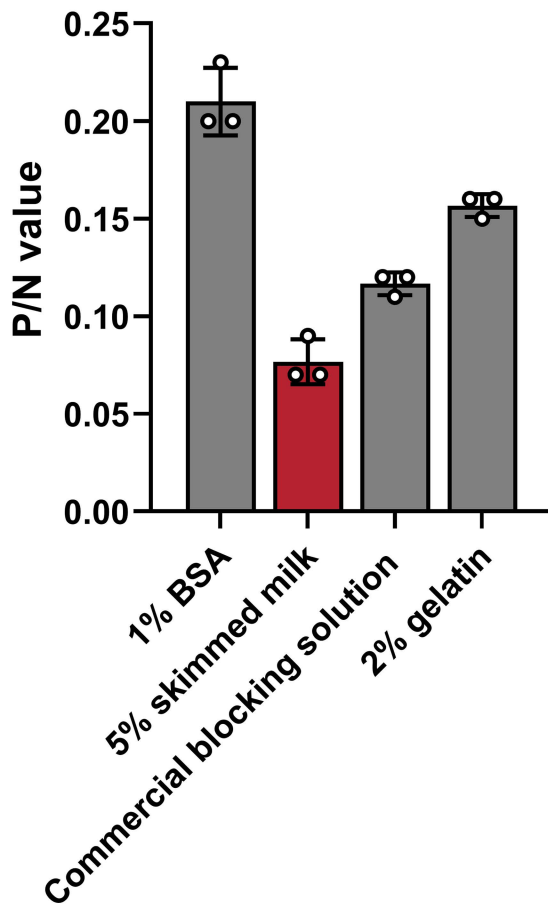**Incubation Time of Antibodies**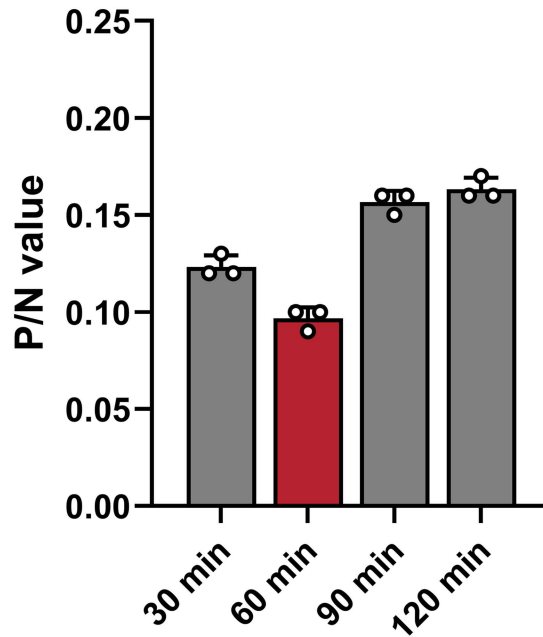**Colorimetric Reaction Time**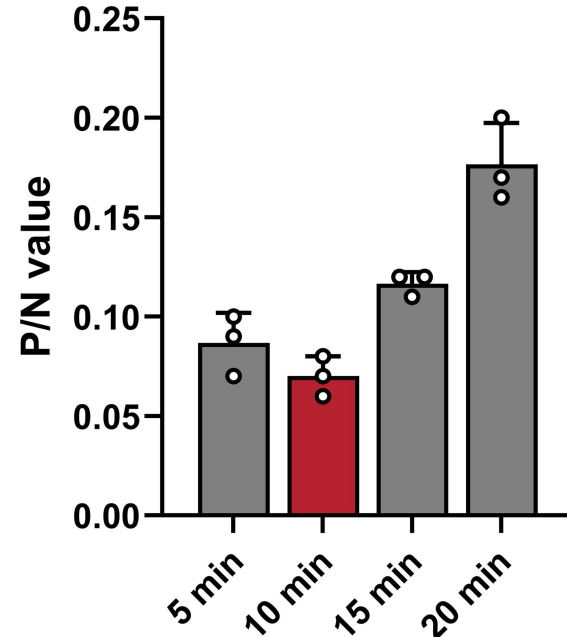

Supplement: Supplementary Figure 1 — Optimization of experimental method conditions for BVDV E2 cELISA. (A) Evaluation of blocking buffers (1% BSA, 5% skimmed milk, commercial blocking solution, and 2% gelatin) for optimization of BVDV E2 cELISA. (B) Evaluation of the incubation time of the sera and HRP-conjugated mAb 3E6 for BVDV E2 cELISA (30, 60, 90, and 120 mins). (C) Evaluation of colorimetric reaction times (5, 10, 15, and 20 mins) for TMB buffer used to optimize BVDV E2 cELISA. [file DataSheet1.pdf]
