# Supplementary material for: Development of a pan-genotypic monoclonal antibody-based competitive ELISA for the detection of antibodies against Bovine viral diarrhea virus
Source: Front Immunol. 2024 Nov 25;15:1504115. doi: 10.3389/fimmu.2024.1504115 (PMC11625775; doi:10.3389/fimmu.2024.1504115)
Supplement: Supplementary file 2 [file Table1.docx]

**Supplementary Table 1**

Optimal E2 antigen coating concentration.

| E2 protein  μg/mL | HRP-conjugated anti-BVDV-E2 mAb 3E6 dilution | | | | |
| --- | --- | --- | --- | --- | --- |
|  | 50 | 100 | 200 | 400 | 800 |
| 2 | 1.598 | 1.478 | 1.328 | 1.145 | 0.771 |
| 1 | 1.564 | 1.436 | 1.288 | 0.932 | 0.74 |
| 0.5 | 1.533 | 1.415 | 1.176 | 0.797 | 0.698 |
| 0.25 | 1.46 | 1.325 | 1.005^a^ | 0.599 | 0.663 |
| 0.125 | 1.387 | 1.254 | 0.795 | 0.363 | 0.538 |
| 0.0625 | 1.165 | 0.932 | 0.538 | 0.153 | 0.273 |

^a^The optimal amount of E2 protein and 3E6 with different dilutions were selected when the OD_450_ value of the direct ELISA was approximately 1.0.
