# Supplementary material for: Development of a pan-genotypic monoclonal antibody-based competitive ELISA for the detection of antibodies against Bovine viral diarrhea virus
Source: Front Immunol. 2024 Nov 25;15:1504115. doi: 10.3389/fimmu.2024.1504115 (PMC11625775; doi:10.3389/fimmu.2024.1504115)
Supplement: Supplementary file 3 [file Table2.docx]

**Supplement Table 2**

Optimal serum dilution for cELISA.

| No. serum | Serum type | 1:1 | 1:2^a^ | 1:4 | 1:8 | 1:16 | 1:32 | 1:64 |
| --- | --- | --- | --- | --- | --- | --- | --- | --- |
| 1 | Positive | 0.104 | 0.118 | 0.176 | 0.281 | 0.282 | 0.548 | 1.03 |
|  | Negative | 0.88 | 1.037 | 1.21 | 1.271 | 1.275 | 1.279 | 1.276 |
|  | P/N | 0.12 | 0.11 | 0.15 | 0.22 | 0.22 | 0.43 | 0.81 |
| 2 | Positive | 0.169 | 0.188 | 0.271 | 0.397 | 0.647 | 0.875 | 1.216 |
|  | Negative | 0.9 | 1.041 | 1.181 | 1.197 | 1.25 | 1.261 | 1.27 |
|  | P/N | 0.19 | 0.18 | 0.23 | 0.33 | 0.52 | 0.69 | 0.96 |
| 3 | Positive | 0.197 | 0.228 | 0.277 | 0.484 | 0.611 | 0.893 | 1.041 |
|  | Negative | 0.86 | 1.098 | 1.142 | 1.223 | 1.26 | 1.25 | 1.306 |
|  | P/N | 0.23 | 0.21 | 0.24 | 0.40 | 0.48 | 0.71 | 0.80 |

^a^The best dilution was selected when the OD_450_ value of positive-to-negative (P/N) sera was smallest.
